# Sleep disturbances and progression of mobility disability: Longitudinal findings from the Nurses’ Health Study

**DOI:** 10.1016/j.sleepe.2023.100071

**Published:** 2023-12-14

**Authors:** Daniel Whibley, Monica M. Shieu, Galit Levi Dunietz, Tiffany J. Braley

**Affiliations:** a Department of Physical Medicine and Rehabilitation, University of Michigan, Ann Arbor, MI, USA; b Institute for Healthcare Policy and Innovation, University of Michigan, Ann Arbor, MI, USA; c Department of Neurology, Divisions of Neuroimmunology/Multiple Sclerosis and Sleep Medicine, University of Michigan Ann Arbor, MI, USA

**Keywords:** Epidemiology, Women’s health, Obstructive sleep apnea, Sleepiness, Aging

## Abstract

**Objective:**

To examine longitudinal associations between self-reported sleep disturbances and mobility disability progression among women, including subgroups with multiple sclerosis (MS), diabetes, and osteoarthritis (OA).

**Methods:**

Prospective cohort study using data from Nurses’ Health Study long-form questionnaires (2008, 2012, 2014, 2016). Logistic regression was used to quantify associations between sleep-related variables at baseline and subsequent increase in mobility disability.

**Results:**

Of 70,303 women (mean age 73), 392 had MS, 7,302 had diabetes, and 24,099 had OA. Between 2008–2016, mobility disability increased by 16.9 % overall, 27.8 % in the MS subgroup, 27.0 % in the diabetes subgroup, and 23.7 % in the OA subgroup. Known/suspected obstructive sleep apnea was significantly associated with an increase in mobility disability between 2008 and 2016, overall (OR:1.4, 95 %CI:1.2,1.5), and in the diabetes (OR:1.5, 95 %CI:1.2,1.9) and OA subgroups (OR:1.2, 95 %CI:1.0,1.4), but not in the MS subgroup (OR:2.3, 95 %CI:0.6,8.9); however, across 2012–2016, this association was significant for MS (OR:4.0, 95 % CI:1.0,16.1). Suboptimal sleep duration was significantly associated with increased odds of mobility disability progression overall, but not in disease subgroups. Perception of adequate sleep was associated with lower odds of mobility disability progression overall (OR:0.82, 95 %CI:0.78,0.87) and for the OA subgroup (OR:0.83, 95 % CI:0.76,0.91). Excessive daytime sleepiness was associated with mobility disability progression overall (OR:1.2, 95 %CI:1.1,1.4) and for the OA subgroup (OR:1.2, 95 %CI:1.0,1.4).

**Conclusions:**

Prevalent sleep disturbances could increase disability progression among women. Chronic disease populations may be uniquely vulnerable. Informed by these data, future research could offer new insight into sleep-based strategies to ameliorate mobility decline.

## 1. Introduction

Impaired mobility is the leading cause of disability in the U.S., affecting an estimated 13 % of the adult population [1]. Prevalence of mobility disability is higher for older adults, women, and racial/ethnic minority groups, who endure loss of independence, increased falls risk, poorer quality of life, higher healthcare services use, and early mortality [1-5]. Although a secular, temporal trend of increase in mobility disability reflects the aging population [6,7], physiological, psychological, and lifestyle factors may also contribute [8]. Identification of modifiable factors could inform interventions to reduce mobility disability and its negative consequences.

Across long-term neurological, metabolic, and musculoskeletal conditions, mobility reductions are observed beyond expected effects of aging [9-12]. For example, in multiple sclerosis (MS), mobility disability may arise from weakness, spasticity, and/or sensory and balance impairments [13]. In diabetes mellitus, mobility disability may be ascribed to poor glycemic control, microvascular changes in the brain and periphery, reduction in muscle mass and lower limb strength [9], and/or sequalae of diabetic peripheral neuropathy [14]. In osteoarthritis (OA), a primarily musculoskeletal condition, mobility disability may arise from pain and stiffness related to cartilage, bone and soft tissue degeneration [10]. While identifying disease-specific factors that contribute to mobility disability is essential, exploration of common treatable contributors is also needed.

Poor sleep health may be one common factor that drives accelerated reduction in mobility among people with chronic conditions. Indeed, studies have demonstrated associations between obstructive sleep apnea (OSA) and both central and peripheral nervous system diseases that could contribute to progressive gait dysfunction [15-19]. Furthermore, sleep deprivation and fragmentation may contribute to chronic pain and exacerbate inflammatory pathways across chronic conditions [20-24]. Yet, despite these demonstrated associations between sleep-related factors and physical and/or functional limitations [25-30], associations between sleep and ambulatory outcomes in populations most vulnerable to mobility disability are underexplored. As sleep disorders and disturbances are largely treatable, insights could offer opportunities to address a driver of physical disability in general and across common chronic conditions.

The objective of this study was to examine longitudinal associations between sleep health and mobility decline necessitating use of assistive devices over 8 years among Nurses’ Health Study (NHS) participants, including subgroups of women with MS, diabetes, and OA. The NHS provides a large cohort with prospectively collected long-term follow-up data from across the US. The size of the cohort supports quantification of associations between indicators of sleep health and mobility disability in general, as well as within disease-specific subgroups. The NHS was purposively designed to establish a homogenous cohort in terms of level of education, health awareness and literacy, supporting collection of high-accuracy self-report data [31]. As an all-female cohort, the NHS provides an excellent resource for sleep epidemiology research given the more frequent reports of poor sleep quality and symptoms of insomnia observed in women when compared to men [32]. We hypothesized that, in the general NHS sample, poorer sleep health would be associated with more rapid decline in mobility, and that effects would be stronger in subgroups defined by exemplar conditions affecting the neurological (MS), metabolic (diabetes), or musculoskeletal (OA) systems.

## 2. Methods

### 2.1. The Nurses’ Health Study

The Nurses’ Health Study (NHS) commenced in 1976 with enrollment of 121,700 female registered nurses aged 30–55 residing in eleven U.S. states. Cohort members were followed up with biennial questionnaires. For this study, we included women who completed an NHS long-form questionnaire in 2008 (baseline) and follow-up questionnaires in 2012, 2014, and 2016 (mobility/assistive device items were not included in the 2010 questionnaire). The NHS cohort study is conducted in accordance with the Declaration of Helsinki [33]. Questionnaire completion implied informed consent. Given the de-identified nature of the dataset, the current study was granted “exempt and not regulated” status by the University of Michigan Institutional Review Board.

### 2.2. Disease cohorts

Disease cohorts (MS, diabetes, and OA) were identified by a positive response to baseline questions about having received a clinician diagnosis.

### 2.3. Daytime sleepiness

Daytime sleepiness was measured using the Epworth Sleepiness Scale (ESS) [34]. Participants were asked to rate their usual chance of dozing or falling asleep while engaged in eight different sedentary activities (4-point response scale; 0=never, 3=high probability). Total scores range from 0 to 24, with ≥10 indicating excessive daytime sleepiness.

### 2.4. Sleep duration

Sleep duration was assessed with the question “On average, over a 24 h period, do you sleep <5, 5, 6, 7, 8, 9, 10+ h. Sleep duration was categorized into <6 (short sleep), 6–8 (reference category), and > 8 h (long sleep).

### 2.5. Adequate sleep

A binary variable was created that reflected responses to the question “Do you feel that your sleep duration is adequate? (yes or no).

### 2.6. Obstructive sleep apnea

Known or suspected OSA was determined by 1) positive endorsement of clinical diagnosis; or 2) responses to six items from a modified STOP-Bang questionnaire (See Table S1 in the supplemental material). The STOP-Bang is an 8-item screener that assesses factors known to confer OSA risk (Snoring, Tiredness, Observed apneas, high blood Pressure, BMI, Age, Neck circumference, Gender) [35]. Item scores are based on yes/no answers, with >5 indicating high risk for OSA. A modified STOP-Bang score which includes 2 positive “STOP” items plus confirmation of male gender and/or BMI>35 has been reported to reflect high OSA risk, and to be more sensitive than total score alone [36]. In the 2008 NHS questionnaire, items were included that were comparable to six of the eight items in the original STOP-Bang and supported OSA risk assessment [37]. High-risk cases were defined by presence of 1) a modified STOP-Bang score ≥3, with at least two positive items from “STOP” items, plus BMI >35^36^; or 2) total modified STOP-Bang ≥4 (any combination of items). This approach has been used in a large-scale epidemiological study [38] and has been shown to be as effective as the original STOP-Bang when self-completed [39].

### 2.7. Mobility disability

In 2008, participants were asked “Do you usually use a cane or walker?”, response options: yes or no. The 2012 survey included the item, “Do you usually use a cane, walker or wheelchair/scooter?”, with response options: no, cane, walker, or wheelchair/scooter. The 2014 and 2016 surveys included the item, “Do you usually use a cane, walker or wheelchair/scooter?”, with response options: no, cane, walker, wheel-chair/scooter, or unable to walk. As more detail was included from 2012 onwards, an increase in use of assistive devices was calculated for two time periods (2008–2016, and 2012–2016). Those who transitioned to a more advanced level of assistance or non-ambulatory status (e.g., cane to wheelchair; wheelchair to unable to walk) were considered positive for mobility disability progression. Cane and walker were collapsed into one category and six women who reported improvement (i.e. reduction in mobility disability) were excluded.

### 2.8. Demographic and health-related variables

Demographic variables were retrieved from 2008 survey responses. Age was calculated from birth year. BMI was calculated as weight divided by height squared (kg/m^2^). Race was categorized as white and non-white. Marital/cohabitation status was classified as married/cohabiting versus other. Current employment status was classified as currently working versus other. Presence or history of osteoporosis, hip replacement, and hip fracture were defined by a positive answer to the question “Have you ever had any of these clinician-diagnosed illnesses or procedures?”.

### 2.9. Statistical analysis

Descriptive statistics were calculated as proportions for categorical variables and means and standard errors for continuous variables. Summary statistics, including sleep-related and assistive device use variables, were calculated for the total population and for those with the chronic conditions of interest. Associations between sleep-related variables were examined using the Pearson correlation coefficient or chi-squared test, and associations between sleep-related variables and mobility disability were examined using logistic regression with adjustment for age, race, BMI, osteoporosis, hip replacement, hip fracture and, for subgroup analyses not constituted exclusively of respondents with a specific diagnosis, diabetes and OA. Due to the small size of the MS subgroup, MS models were adjusted for age only. All analyses were conducted using SAS 9.4 (SAS Institute Inc, Cary, NC).

## 3. Results

### 3.1. Baseline descriptive statistics

Data from 70,303 nurses were available for analysis. Mean age at baseline was 73, with 329 endorsing an MS diagnosis, 7,302 diabetes, and 24,099 OA. Compared to nurses without MS, those with MS were more likely to be younger, married, and less likely to be working. Compared to those without diabetes, those with diabetes were more likely to be older, and less likely to be married or working. Nurses with OA were more likely to be younger, and less likely to be married or working when compared to those without OA (Table 1).

### 3.2. Baseline descriptive statistics for sleep-related variables

Table 2 shows descriptive statistics for sleep-related variables in 2008. Overall, prevalence of known/suspected OSA was 7.0 % and was significantly higher among diabetes (15.7 %) and OA subgroups (9.4 %), but not MS (6.4 %). However, respondents with MS had indication of poorer sleep health across all other sleep-related variables when compared to those without MS. A similar pattern of poor sleep health was observed among diabetes and OA subgroups when compared to their non-disease counterparts (Table 2).

### 3.3. Associations between sleep-related variables at baseline for the total sample

Women with known or suspected OSA had a higher frequency of longer sleep duration (>8 h) compared to those without known or suspected OSA (17.68 % vs 11.11 %, chi-squared (2, *N* = 69,734)=236.48, *p* < 0.0001) and more frequently reported feeling that their sleep duration was inadequate (44.03 % vs 30.48 % for those without known or suspected OSA, chi-squared (1, *N* = 69,278)=381.21, *p* < 0.0001). Also, a higher proportion of those with known or suspected OSA crossed the ESS threshold for excessive daytime sleepiness (36.21 % vs 6.30 % for the group without known or suspected OSA, chi-squared (1, *N* = 70,105)=5288.90, *p* < 0.0001).

There was a small, positive correlation between sleep duration and ESS score (Pearson correlation coefficient=0.04, *p* < 0.0001). Compared to the group who reported that their sleep duration was inadequate, those who reported that they felt that their sleep duration was adequate had a higher proportion in the 6–8 h sleep duration category (70.13 % vs 29.87 %, chi-squared (2, *N* = 68,883)=6067.52, *p* < 0.0001). Among the group with excessive daytime sleepiness, there was a higher proportion of those with long sleep (>8 h) (13.93 % vs 9.53 % of this subgroup who reported sleeping <6 h and 7.47 % reporting sleep duration of 6–8 h, chi-squared (2, *N* = 69,664)=392.68, *p* < 0.0001). A lower proportion of those with excessive daytime sleepiness reported feeling that their sleep was adequate when compared to those without excessive daytime sleepiness (6.91 % vs 93.09 %, chi-squared (1, *N* = 69,215)=404.97, *p* < 0.0001).

These findings are expected and likely reflect mediating pathways between the sleep-related variables (e.g., longer sleep duration and excessive daytime sleepiness are established downstream consequences of OSA). Given these results and plausible causal associations between the different sleep-related variables, we did not mutually adjust for sleep-related variables in a single model to avoid Table 2 Fallacy [40].

### 3.4. Prevalence of onset or increase in mobility disability

Table 3 displays data on assistive device use/ambulatory status, with proportions for increase provided for 2008–2016 and 2012–2016 periods. Overall, mobility disability increased by 16.9 % between 2008 and 2016, and by 11.6 % between 2012 and 2016. MS, diabetes, and OA subgroups had a higher increase in assistive device use for both periods when compared to those without these diseases.

### 3.5. Associations between sleep variables and increase in mobility disability

Associations between sleep variables and increase in mobility disability are provided in Table 4. Known/suspected OSA was associated with increase in mobility disability between 2008 and 2016 overall (OR 1.4, 95 % CI 1.2–1.5), and in diabetes (OR 1.5, 95 % CI 1.2–1.9) and OA subgroups (OR 1.2, 95 % CI 1.0–1.4), with the largest (but not statistically significant effect) for the MS subgroup (OR 2.3, 0.6–8.9). Known/suspected OSA was also associated with increased odds of mobility disability progression between 2012 and 2016 in the total population (OR 1.3, 95 % CI 1.2–1.5) and in all disease subgroups. Again, the largest effect was observed in the MS subgroup (OR 4.0, 95 % CI 1.0, 16.1). The effect in the diabetes subgroup was comparable to the total sample (OR 1.3, 95 % CI 1.0–1.7), and the effect in the OA subgroup was slightly smaller and not statistically significant (OR 1.2, 95 % CI 0.99–1.4) (Table 4a).

Sleep of short or long duration was associated with increased odds of mobility disability progression between 2008 and 2016 in the total sample when compared to report of 6–8 h of sleep. Larger mobility disability effects were observed for sleep of longer duration and were also observed for the 2012–2016 period. For all disease subgroups and follow-up periods, short or long sleep duration was not associated with assistive device use escalation. (Table 4b).

Perception of adequate sleep was associated with lower odds of mobility disability progression between 2008 and 2016 in the total sample (OR 0.82, 95 % CI 0.78–0.87) and in the OA (OR 0.83, 95 % CI 0.76–0.91) but not diabetes or MS subgroups. (Table 4c).

Excessive daytime sleepiness was associated with increase in mobility disability progression between 2008 and 2016 overall (OR 1.2, 95 % C1.1–1.4) and for the OA subgroup, but not the MS or diabetes subgroups. Similar associations were seen for the 2012–2016 period (Table 4d).

## 4. Discussion

Mobility disability is a leading public health challenge that disproportionately affects older adults, minoritized groups, and people with chronic conditions. Prevention, or more realistically, delayed onset to ambulation impairment, may be achieved if contributing factors are identified and mitigated. In this cohort of U.S. women, we focused on sleep health as an under-recognized contributor to mobility disability. We hypothesized that poor sleep health would be associated with escalation in assistive device use or transition to non-ambulatory status over an eight-year follow-up period, and that associations would be stronger among subgroups of women with exemplar diseases affecting neurological, metabolic, or musculoskeletal systems.

Some, but not all, sleep-related factors were associated with onset or increase/escalation in assistive device use. Known/suspected OSA was consistently associated with mobility disability progression, overall and within disease subgroups. Although not statistically significant across the 8-year follow-up period, the magnitude of effect was strongest among women with MS and was significant over the latter half of the follow-up period. Given the smaller size of the MS subgroup, it is likely that the non-significant findings can be attributed to insufficient power to detect ‘true’ effects; however, confirmation in a larger or more disabled MS cohort is required. Indeed, prior investigations have identified associations between OSA and microvascular changes in the brain and periphery, which could plausibly affect systems associated with gait.

For other sleep health indicators, contrary to expectations, we also identified strong associations with increased assistive device use among those *without* exemplar diseases. Overall, there was an association between suboptimal sleep duration and increase in assistive device use not seen in the MS, diabetes, or OA subgroups. Notably, we categorized 6–8 h of sleep as optimal. As the U.S. National Sleep Foundation recommends 7–8 h for older adults [41] the increased leniency of our categorization could at least in part explain the non-significant association between sleep duration and assistive device use increase in disease subgroups. However, it is also possible that sleep duration, assuming it accurately represents the individual’s experience in the NHS, requires context and, coherent with arguments for multidimensional sleep phenotyping [42-44], that information about timing, rhythmicity, and regularity of sleep may be necessary to characterize an individual’s sleep pattern. Given differential findings for those without versus with the exemplar diseases in this study, it is possible that a more nuanced and contextual understanding of sleep duration and timing is of greater importance for people living with chronic conditions.

There was a positive association between excessive daytime sleepiness and increase in mobility disability, and between perception of adequate sleep and lower likelihood of mobility decline overall and in the OA subgroup, but not for MS and diabetes subgroups. These results run somewhat contrary to classically described associations between excessive daytime sleepiness and perception of inadequate sleep and OSA, which was in and of itself a predictor of increase in assistive device use in disease subgroups in our study. Examination of the validity of single-item sleep questions and sensitivity/potential for misclassification using limited responses options is warranted. However, despite potential measurement imprecision, overall, our findings suggest that sleep health is an important contributor to mobility disability regardless of presence of a chronic disease, but for people with chronic diseases, and potentially for those with MS specifically, identification and effective management of OSA should be a priority.

A large number of factors have been associated with mobility disability in earlier studies; however, within both general and systematic reviews, sleep-related factors have not been included [8,45]. Furthermore, a 2021 meta-analysis that focused specifically on sleep disturbances and physical impairment only identified three prospective studies that examined mobility as an outcome [30]. Poor sleep quality was associated with higher likelihood of mobility disability in community-dwelling adults aged >60 [28]; higher score on a summary dyssomnia measure was associated with a 27 % higher risk of incident mobility disability in older adults [29]; and frequent sleep disturbances were associated with mobility impairment among adults with mean baseline age of 50 [46]. Importantly, measures of mobility disability used in these studies did not incorporate information about use of assistive devices, with items instead focused on self-reported difficulties executing functional tasks.

Operationalization of our mobility disability outcome relied on self-report of assistive device use. Within a clinical context, mobility assessments routinely include examination of gait, balance, and transfers, with limitations or impairments guiding assistive device provision. A range of instruments are available to support evaluation, with the majority permitting assistive device use during administration [47]. Use of mobility modifications, including use of an aid was identified as one of three factors associated with mobility decline with moderate-to-large effects in a 2021 systematic review (alongside older age and wide-spread pain) 0 [45]. This provides some support for our use of assistive device as a proxy for increase in mobility disability. However, comparison between self-report and objective mobility measures have revealed moderate correlation [48]. Performance-related tests, e.g., the Timed-Up-and-Go, are generally conducted in standardized, clinical environments. Our outcome measure could be argued to have better ecological validity, however, we have no data on cumulative/duration of device use, or any broader context that integrates interpersonal and/or environmental factors as barriers and/or facilitators of mobility (see Yeom, Fleury and Keller for a social-ecological framework of mobility limitations in older adults [8]).

Notable study strengths are the sample size provided by the NHS dataset, which facilitated disease/system-based comparative analysis, the longitudinal follow-up period (enabling assessment of temporality), comparison of follow-up periods of different lengths, and inclusion of items that supported population-level OSA risk screening (an important feature to capture undiagnosed cases, as approximately 90 % of women with OSA remain undiagnosed [49]).

### 4.1. Study limitations

Although the NHS cohort provided a large sample size with very low attrition, compared to the general population, this cohort has a slightly higher educational and socioeconomic status, is mostly white, and is exclusively comprised of women. Although this limits generalizability of our findings, restricting the sample to nurses has been argued to enhance internal validity due to the assumed high level of health knowledge and literacy, and commitment to health-related knowledge generation, potentially explaining high levels of participant retention and data completeness [50].

Given the perennial concern of confounding in observational research, we carefully considered the available variables that we could adjust for in our models, with attention given to inclusion of markers of diet quality, leisure-time physical activity, and sedentary behavior. However, given the extant literature on prospective associations between parameters of sleep and subsequent food choice [51], and sedentary or physical activity/exercise behavior specifically in older women [52], as well as probable consequential effects of these variables on disability-related variables, it is highly likely that these factors lie on the causal path between poor sleep and subsequent mobility disability progression. It is also plausible that sleep-related factors and mobility disability are independent risk factors for diet quality, leisure-time physical activity, and sedentariness (i.e., a collider effect may be activated by their adjustment). Our decisions regarding adjustment variables were underpinned by the concept of Table 2 Fallacy [40], which also informed our decision not to mutually adjust for all sleep-related variables in a single model.

### 4.2. Future directions

Our findings provoke research questions for future enquiry: Would the associations hold true for men and, if so, would the magnitude of effects be comparable? It is possible that findings in men could be attenuated since women have been observed to experience faster decrease in muscle strength with increasing age [53]. Is the impact of sleep health on disability mediated through its effect on other factors (including disease-related factors such as inflammation, or symptoms such as pain and fatigue)? A carefully designed mediation analysis using a causal framework would help to shed light on the potentially pivotal importance and underlying mechanisms linking sleep health and mobility disability. An additional and important area of enquiry would be to examine the influence of social determinants of health as moderators of the effects of sleep health on mobility disability. Our findings provide the foundation for further research in this area and support continued consideration of indicators of sleep health as part of a constellation of factors contributing to mobility disability.

## Supplementary Material

Table S1: High OSA risk corresponding to STOP-Bang questionnaire items

## Figures and Tables

**Table 1:** Baseline demographics.

	Total (*N* = 70,303)	Multiple sclerosis subgroup (*N* = 329)	Diabetes subgroup (*N* = 7,302)	Osteoarthritis subgroup (*N* = 24,099)
Age (years), mean (SE)	73.1 (6.8)	70.5 (6.3)	73.9 (6.5)	59.0 (4.3)
Race/ethnicity				
white (%)	96.7	97.9	94.5	97.1
Black (%)	1.4	0.6	2.7	1.2
Hispanic (%)	0.9	1.5	1.2	0.8
Other (%)	1.1	0.0	1.6	0.9
Married or cohabitating (%)	63.5	67.7	58.6	61.5
Currently working (%)	20.3	14.1	15.3	15.5

SE: Standard Error.

**Table 2:** Prevalence of sleep-related variables at baseline.

	Total (*n* = 70,303)	Multiple sclerosis subgroup (*N* = 329)	Diabetes subgroup (*N* = 7,302)	Osteoarthritis subgroup (*N* = 24,099)
**Sleep apnea**				
Sleep apnea diagnosis (%)	3.9	3.7	9.0	5.7
High OSA risk^a^ (%)	3.7	3.7	9.0	4.7
Known or suspected OSA^b^ (%)	7.0	6.4	15.7	9.4
**Sleep duration**				
< 6 h (%)	6.6	8.0	8.3	7.8
6–8 h (%)	81.8	73.4	77.1	80.4
> 8 h (%)	11.6	18.7	14.6	12.5
**Adequate sleep (%)**	68.6	64.0	63.5	64.0
**Excessive daytime sleepiness (%)** ^c^	8.4	11.0	12.7	9.5

OSA=Obstructive Sleep Apnea.

a Defined by modified STOP-Bang score.

b Defined by either having a OSA diagnosis or high OSA risk by modified STOP-Bang score.

c Defined by Epworth Sleepiness Scale score.

**Table 3:** Prevalence of onset or increase in mobility disability.

	2008–2016 (%)	2012–2016 (%)
Total sample (*n* = 70,303)	16.9	11.6
Multiple sclerosis subgroup (*n* = 329)	27.8	17.8
Diabetes subgroup (*n* = 7302)	27.0	17.9
Osteoarthritis subgroup (*n* = 24,099)	23.7	15.2

**Table 4:** Associations between sleep-related variables and increase in mobility disability.

A. Exposure: obstructive sleep apnea	Time period 2008–2016 OR (95 % CI)	Time period 2012–2016 OR (95% CI)
Total sample^a^	1.4 (1.2, 1.5)	1.3 (1.2, 1.5)
Multiple sclerosis subgroup^b^	2.3 (0.59, 8.9)	4.0 (1.0, 16.1)
Diabetes subgruop^c^	1.5 (1.2, 1.9)	1.3 (1.0, 1.7)
Osteoarthritis subgroup^d^	1.2 (1.0, 1.4)	1.2 (0.99, 1.4)
B. Exposure: sleep duration	Time period 2008–2016 OR (95 % CI)	Time period 2012–2016 OR (95 % CI)
Total sample^a^		
<6 h	1.1 (1.0, 1.3)	1.1 (0.94, 1.2)
>8 h	1.2 (1.1, 1.3)	1.2 (1.1, 1.3)
Multiple sclerosis subgroup^a^		
<6 h	0.44 (0.09, 2.1)	1.3 (0.34, 5.1)
>8 h	0.71 (0.28, 1.8)	0.49 (0.14, 1.7)
Diabetes subgroup^c^		
<6 h	1.3 (0.96, 1.7)	0.96 (0.69, 1.3)
>8 h	1.2 (0.95, 1.5)	1.2 (0.89, 1.5)
Osteoarthritis subgroup^d^		
<6 h	1.1 (0.92, 1.3)	1.0 (0.86, 1.3)
>8 h	1.1 (0.95, 1.2)	1.1 (0.91, 1.2)
C. Exposure: Perception of adequate sleep duration	Time period 2008–2016 OR (95 % CI)	Time period 2012–2016 OR (95 % CI)
Total sample^a^	0.82 (0.78, 0.87)	0.88 (0.82, 0.94)
Multiple sclerosis subgroup^b^	1.0 (0.51, 2.0)	0.89 (0.4, 2.0)
Diabetes subgroup^c^	0.90 (0.76, 1.1)	0.91 (0.76, 1.1)
Osteoarthritis subgroup^d^	0.83 (0.76, 0.91)	0.90 (0.82, 0.99)
D. Exposure: Excessive daytime sleepiness	Time period 2008–2016 OR (95 % CI)	Time period 2012–2016 OR (95 % CI)
Total sample^a^	1.2 (1.1, 1.4)	1.2 (1.1, 1.3)
Multiple sclerosis subgroup^b^	0.71 (0.19, 2.7)	0.38 (0.05, 3.0)
Diabetes subgroup^c^	1.2 (0.96, 1.6)	1.2 (0.90, 1.6)
Osteoarthritis subgroup^d^	1.2 (1.0, 1.4)	1.1 (0.91, 1.3)

OR: Odds Ratio.

CI: confidence interval.

a Adjusted for age, race (white vs non-white), diabetes, osteoporosis, osteoarthritis, hip replacement, hip fracture, BMI.

b Adjusted for age.

c Adjusted for age, race (white vs non-white), osteoporosis, osteoarthritis, hip replacement, hip fracture, BMI.

d Adjusted for age, race (white vs non-white), diabetes, osteoporosis, hip replacement, hip fracture, BMI.
