# Supplementary material for: Sleep disturbances and progression of mobility disability: Longitudinal findings from the Nurses’ Health Study
Source: Sleep Epidemiol. Author manuscript; Available in PMC 2025 Jan 16. (PMC11737507; doi:10.1016/j.sleepe.2023.100071)
Supplement: Table S1: High OSA risk corresponding to STOP-Bang questionnaire items [file NIHMS2041525-supplement-1.docx]

**Supplemental 1**

| Questions from the NHS file | Cutoffs of the NHS questions considered as a positive result | Corresponding STOP-Bang item |
| --- | --- | --- |
| How often do you snore? | Every night, most nights | Do you snore loudly? |
| Epworth sleepiness scale | ≥10 | Do you often feel tired, fatigued, or sleepy during the daytime? |
| N/A | N/A | Has anyone observed you stop breathing during sleep? |
| Have you ever had a high blood pressure diagnosis?  Current usual blood pressure | Yes  Systolic>124 mmHg, or diastolic>74 mmHg | Do you have (or are you being treated for) high blood pressure? |
| BMI | >35 kg/m^2^ | BMI |
| Year born | >50 years | Age |
| N/A | N/A | Neck circumference |
| Sex | Men | Sex |

NHS: Nurse’s Health Study

**Table S1: High OSA risk corresponding to STOP-Bang questionnaire items**
